# Annual Commencement of Niagara University

**Published:** 1887-05

**Authors:** 


### Editorial.

### *ANNUAL COMMENCEMENT OF NIAGARA UNIVERSITY.*

### MEETING OF ALUMNI ASSOCIATION—THE CONVOCATION—THE BANQUET.

The annual meeting of the Alumni Association and commencement exercises of Niagara University Medical Department was held at the college building on Ellicott street in this city. The building, which was erected three years ago, was in fine order, and many expressions of surprise were heard at the unusual neatness, "for a medical college." The Board of Curators arrived promptly at 10 A. M., and organized.

There were present Drs. F. W. Ross and Henry Food, Elmira; Drs. Granger, W. W. Potter, M. Hartwig, C. A. Gould, Buffalo. It was learned that Dr. Banta, of Buffalo, Dr. Carroll, of Rochester, and Dr. Gregory Boyle, of Syracuse, were unavoidably detained. At 11 A.M., the Alumni held its meeting; Dr. Wm. H. Heath, President, formerly a professor in this college, occupied the chair. Dr. Lewis, Secretary, and Dr. Murphy, First Vice-President, were at their posts. Dr. Heath delivered the President's address. He referred to the progress the college had made, complimented the faculty on the high standard of education maintained, reminded the Alumni of the high mark the faculty had placed for them, and hoped many would reach it, after which officers were elected as follows:

*President*, Dr. Floyd S. Crego; *First Vice-President*, Dr. E. J. Murphy; *Second Vice-President*, Dr. C. C. Fredericks; *Permanent Secretary*, Dr. George W. T. Lewis, corner Utica street and Linwood avenue; *Treasurer*, Dr. Henry D. Ingraham; *Executive Board*, Dr. Frank H. Potter, Buffalo; Dr. Simeon T. Clark, Lockport; Dr. Charles G. Stockton, Buffalo.

Dr. Stephen Smith, of New York, was elected an honorary member. Seven new names were, later in the day, added to the list of members—four from the present class, three from former classes.

There was a large number of medical men present at the afternoon session, the lower amphitheatre being more than filled. Dr. Simeon T. Clark delivered the address of welcome, which was couched in elegant and appropriate language. Dr. Floyd S. Crego followed with a paper on defective mental inhibition, with loss of consciousness and all recollection. He reviewed the functions of the brain and their mode of action; told how and when these parts lost their integrity; what could be disease of the brain, and what a condition simply. He proved there was a mental inhibition function, and hence a mental inhibition center. He described cases coming within his own practice and reported in the text-books where there was loss of control; where patients had committed crimes without apparent motive, and when, after the deed, not a trace of mental disturbance was apparent. He called the attention of the physicians to the necessity of obtaining correct histories of their patients. He said lawyers and medical men who are called as medico-legal witnesses, who have to weigh the social and legal aspects of his opinions, are still chary of admitting loss of control or morbid impulse as an excuse for crime. He cited cases in which there was no difficulty in arriving at a conclusion, and again others where we were confronted with a problem which, in the light of our present knowledge of mental states, we were enabled to solve. He thought with constant study, and with the light of the recent developments and advances in cerebral localization, the time was not far distant when such cases could be explained and classified. The event of the day was the scholarly address of Dr. Stephen Smith, of New York, professor of surgery in the New York University Medical College, author of the standard work on operative surgery, "The Influence of Modern Surgery on Human Longevity." He started out with the remark that life on this planet is maintained only by the most persistent struggle against hostile, outside and inherent causes. On all sides the elements of generation were supplied in a prodigality which in itself proved how uncertain was the very act of securing existence at all. The summer and autumn air swarmed with germs seeking in vain for a soil in which to germinate. The human female was born with a store of 30,000 germs with which to propagate her kind, and with that number barely five on an average, even under the most favorable conditions, attained to actual existence as independent beings. Of this latter number, scarcely two lived to propagate their species. The great aim and purpose of all life was first to secure and second to maintain a foothold in the incongenial soil of this earth. Every individual was found desperately engaged in a struggle, first, to maintain its existence, and second, to increase its species. The severity and success of that struggle depended upon its environments and physical constitution. Generally speaking, the agencies tending most powerfully to abridge life were two-fold: 1, change in the environments unfavorable to the physiological necessities; and 2, the law of succession of organic life, according to which one form subsists upon another. The speaker then referred to man's capacity for long life, the causes which abridge human life, and the value of the methods of practice of modern surgery in protecting man from agencies most fatal to his existence. The question of the length of life was said to be determined by the amount of vital endowment and vital expenditure. Speaking of the normal longevity of man, it was stated that the study of the development of the teeth and of the bones indicated one hundred years as the normal period of human existence. This scientific deduction was confirmed by observation and tradition, and might be accepted as the length of life to which every man has an inherent right to attain. Every death short of that period was due to unnatural or abnormal conditions. Yet the census showed upward of 492,000 deaths in this country in a single year, of whom more than 110,000 were under one year old, while a little less than half the total died under five years. Only 11,000 exceeded seventy, and only 1,300 reached ninety-five and upward. This result was ascribed not to hereditary causes, but to preventible disease, unhealthy surroundings and improper living.

Continuing, he said:

It is scarcely a score of years since the people of England proposed to the Prime Minister to meet an invasion of cholera by fastings and prayers. His reply began a new era in the history of preventive medicine. First, he said, destroy all kinds of sources of filth, and render your homes and their surroundings clean and pure, and then ask the Almighty to bless your efforts. The advice was followed, and cholera passed by England as harmlessly as did the angel of death the marked houses of the Israelites on the Passover night. This may be considered as the first official promulgation of the results of scientific investigations into the real sources of sickness and death among the people. The value of the discovery of the immediate nature of the zymoses to human longevity was chiefly in the direction of providing measures of prevention. They were proved to be preventable diseases, and the saving of life through the medium of sanitary measures has been incalculable. In communities where it has been estimated approximately, the reduction of sickness and death by these pestilences has been from one-third to one-half.

But this great discovery has not, until recently, had any decided influence upon the curative art. Indeed, so far as regards the modern practice of medicine, its influence has been most decided conservatism. The therapeutist has become less and less inclined to resort to positive medication, and more and more disposed to put his trust in the resources of nature. The physician of the present day may well be represented as sitting mutely and reflectively at the bedside, with his finger upon his lips, studying the natural history of the disease, and guarding carefully against any complication, lest that history should be vitiated.

If, on the contrary, we turn to the other great department of the healing art, modern surgery, we can but be amazed at the inspiration which it has caught from the revelations of science in regard to the nature of zymotic diseases. The moment that the natural history of micro-organisms was discovered, the genius of surgery saw its opportunity. As a general, long encamped about the besieged city, suddenly rushes upon the citadel with huge clamor when he learns the clews that lead to it, so the surgeon of to-day, enlightened by science, assumes an aggressive attitude towards disease and boldly attacks it in its strongholds. And what daring he exhibits; what feats he daily accomplishes! Everywhere, and in every land, the air is full of startling rumors of the triumphs of surgery. The medical periodicals teem with reports of new operations, or old and formerly fatal operations now performed with uniform success. Not content with its former position as the handmaid of medicine, surgery is now dominant, and daily makes incursions into the domain of allied departments of practice. Already it has absorbed the entire field of gynecology, largely that of abdominal and hepatic affections and to-day it is making cautious explorations of lung and brain diseases. Where it will ultimately fix the limits and boundaries of its kingdom, no one at this date can predict.

It will be proper to notice, in this connection, the basis on which modern surgery has begun to rear the temple which will, in all time, commemorate its claims as the greatest conservative power of human health and longevity which has as yet appeared among the arts of men.

As has been stated, the discovery of the nature of zymotic diseases was at first useful only in proving that they were preventable by human efforts, rightly directed. Sanitary measures did reduce sickness and death rates, but the discovery did not materially aid the art of curing by the use of therapeutical remedies. It was not until the peculiar action of these micro-organisms in the living tissues became known, and the methods of destroying them were discovered, that it was made apparent that a better knowledge of zymotic diseases would avail materially in actual practice. This knowledge was the result of the long and patient investigations of Sir Joseph Lister, to whom mankind will be forever indebted as to its greatest benefactor. Mr. Lister discovered that micro-organisms can grow in the animal body, giving rise to a variety of diseases; some are fatal to most animals, as the bacillus of anthrax; others are only pathogenic in certain species of animals. The diseases caused by the propagation and growth of these bodies in the blood and tissues are grouped together under the term infective diseases. Of these there are are two kinds, those in which the infection occurs from a wound or open surface—traumatic infective diseases—and those in which no wound is necessary and where the pathogenic organisms are supposed to be able to enter the body through uninjured surfaces. It logically followed that infectious diseases, whether traumatic or idiopathic, could be prevented by protecting the system from the entrance of micro-organisms. This can be effected only by excluding them from the body. To accomplish this object, we may protect the system, or we may destroy the micro-organisms. This is antiseptic surgery, pure and simple. It is also the essence of sanitary science. Aseptic surgery is then based on the principle of the exclusion of micro-organisms from wounds. It is not treatment by spray, nor by gauze, nor by spray and gauze, nor by carbolic acid, but is any method of treatment which aims at, and succeeds in, excluding the causes of suppuration from wounds. And this result is now attained with the greatest certainty. Putrefactive suppuration is excluded from the category of wound complications of the surgeon who faithfully and intelligently employs the recognized means of excluding from wounds the germs of infection.

Let us apply these established facts to the subject under consideration—the influence of modern surgery upon longevity. This we can do only approximately, for as yet statistical results have not been sufficiently recorded. But we may gain a faint perception of the enormous aggregate of life-saving which will in the future accrue when antisepsis in surgery becomes universally adopted and practised, by reviewing some of its gathered fruits from the fields of observation now thoroughly cultivated.

In all of the older works on surgery, we are taught that inflammation is a necessary consequence of the exposure of the surfaces to the air. In contrasting subcutaneous wounds, Hunter remarks: The injuries of the first division, in which the parts do not communicate externally, seldom inflame; while those of the second commonly both inflame and suppurate." Paget remarks: "What Mr. Hunter said is true, especially in wounds in our own bodies; subcutaneous wounds seldom inflame; open wounds generally both inflame and suppurate." He adds: "In these sentences Mr. Hunter has embodied the principle on which is founded the whole practice of subcutaneous surgery; a principle of which, indeed, it seems hardly possible to exaggerate the importance. For, of the two injuries inflicted in a wound—the mechanical disturbance of the parts, and the exposure to the air of those that were covered—the exposure, if continued, is the worse. Both are apt to excite inflammation, but the exposure excites it most certainly, and in the worse form, *i. e.*, in the form which most delays the process of repair, and which is most apt to endanger life." Paget adds, "What is wanted for immediate union is, not a certain undefined, slight degree of inflammation, but the complete absence of inflammation." In former times, Paget states that "after the majority of such wounds as are made in surgical practice, changes ensue, both local and constitutional, which are not necessary parts of the healing process, but rather hindrance to it."

The amount of sickness and the loss of life from wounds which suppurated prior to the antiseptic period, cannot be estimated. If we admit the truth of the assertion of those eminent authorities, inflammation and suppuration, in forms "most apt to endanger life," were the rule in open wounds, which comprise, always, nearly the entire list. Suppuration in wounds, we now know, involves the liability to exhaustion, erysipelas, hectic fever, septicæmia, pyæmia, abscesses, sloughing, gangrene, phagedena, waxy degeneration. More exactly, of 100 wounds which did not involve important parts, 95 suppurated; of the latter number, 23 were accompanied by abscesses; 20 by septicæmia; 5 by pyæmia. Death occurred as a result of suppuration in 15 cases.

Under antiseptic treatment, putrefactive suppuration of wounds is practically an impossibility. Every surgeon who thoroughly understands and fully practices antisepsis, knows that suppuration in a wound under his care shows neglect in the application of the preventive measure. Bilhatte says that "Failure in the treatment of wounds at a surgical clinic, systematically carried on, has become as rare as accidents on a well-managed railroad." Practically, then, suppuration may be excluded from wounds. With this postulate, let us examine more specifically into the results of antiseptic surgery applied under various conditions.

Compound fractures, which do not require immediate amputation, were spoken of by older surgeons as beset with complications, as erysipelas, pyæmia, hectic, exhausting suppuration, necrosis, and viscious position of the broken portions of bone—their management demanding constant and untiring attention to details, etc. And even this statement but faintly describes to the surgeon of that period his labors and anxieties. But now all is changed. Instead of being the objectionable and proscribed cases of the ward, they belong to the more interesting class of accidents. The wound once antiseptically cleansed and antiseptically dressed gives no further trouble. Amputation for compound fractures was very frequent with older surgeons, as compared with the present. In one year, (1851,) twenty amputations were performed in a large hospital for these injuries. In 1886, amputation is rarely performed for these fractures.

He then took up the results of many surgical operations under the old, now under the modern, surgical methods. As to the operations on the uterus, he said:

Cancer of the uterus was formerly a fatal disease. It has been stated that "The death rate from cancer has increased during the last ten years; that three-fourths of all cancers are among women; that one-half of all cancers are cancers of the uterus, and that cancer of the uterus comprises, in Berlin, from one-fourth to one-third of all gynecological complaints. This increased mortality, with the comparatively greater frequency of the diseases among women, and its predilection for the uterine tissues, make extirpation of the uterus for cancer a subject of superior importance at the present time." The success of extirpation has progressively increased during the last five years. In 1880, sixty-three per cent. of all of the operations recovered, while in 1885, the recoveries had risen to seventy-six per cent. of all of the reported operations. But in the hands of the more experienced operators, the recoveries have equaled the more simple operations. For example, Fritsch had ninety-two per cent. of recoveries in twenty-four consecutive cases, and Martin had ninety-one per cent. of recoveries in fifty-five consecutive cases. Finally, Staude has had no death in sixteen consecutive cases. Thus the climax of complete success has been finally reached in one of the most fatal diseases of women.

Like all malignant diseases, cancer of the uterus is liable to return after extirpation of the organ, but the frequency of return is apparently diminishing. Of those cases which have been followed, twenty per cent. were known to have been well at the end of eighteen months. It is believed that recurrence is most general within the first year of the operation; hence, cases which remain well at the end of eighteen months or two years are commonly considered cured. These figures would, therefore, give a clear total gain of life in cancer of the uterus of twenty per cent. of all cases of extirpation which survive the operation; and as the mortality is but eight to ten per cent. in the hands of the best operators, twenty per cent. of complete recoveries may be set down as the degree of success at present attained.

In conclusion, it was stated that human longevity was yet greatly to be extended in this country: (1) through the influences of civilization, which is rendering life free from excessive vital expenditure; (2) through the universal administration of sanitary laws, which will largely prevent foreign and domestic pestilence; (3) through the direct power of antiseptic surgery to remove or destroy the prime factors of the larger number of human maladies.

This brief and imperfect synopsis does not do credit to his masterly effort, at the conclusion of which the speaker received a perfect ovation from those present.

Dr. B. H. Daggett, of Buffalo, exhibited for the first time in Western New York an apparatus by which gaseous enemata were given for the relief of consumption. He exhibited a patient who had taken the gas three days, who was greatly benefited. He presented a report, which appears in this number of the JOURNAL.

Dr. Henry D. Ingraham presented a paper on intubation of the larynx, but owing to the lateness of the hour it was read by title. It will appear in the next issue of the JOURNAL.

Dr. F. H. Potter presented a paper which appears in this number of the JOURNAL.

### THE COMMENCEMENT.

The commencement exercises were held in Association hall, which was filled with a large audience. Wahle's orchestra occupied a conspicuous place in the gallery and enlivened the proceedings. On the platform, which was tastefully decorated with flowers and potted plants, were the Rt. Rev. S. V. Ryan, Chancellor of the University; the Rev. P. V. Kavanaugh, President of the University; the Rev. Father Kircher, Vice-President of the University. The members of the faculty were also present, wearing their university gowns and hoods.

Bishop Ryan, in opening the proceedings, said it was very gratifying to him to be present and to know that those upon whom degrees were to be conferred were worthy of the honor and well prepared for the grave duties and responsibilities devolving upon them. He knew of no profession that stood higher in the estimation of the community than the medical profession. He congratulated the faculty on the success that had attended their labors and on the brightness of the prospects of the university with which they were identified. It spoke well, he thought, for the capacity of the graduates that they had not only been able to pass through the strict examination of the faculty, but also to pass the ordeal of examination by distinguished physicians from different parts of the country. The Right Reverend speaker also referred to the organization of a legal department, and the probability of a college dentistry being established in the near future as a branch of their university. He trusted that all would work harmoniously for the general good of the society. It was in no spirit of rivalry that they went about this work, but for the higher education of those who were to come after them, and he had no doubt they would all try to honorably discharge the duties expected of them. He congratulated the graduates and professors on the good showing presented.

The candidates for the degree of M. D. were: Charles Ellsworth Congdon, Middleport, N. Y.; David Lawrence Redmond, Rochester, N. Y.; Lawrence George Hanley, Mt. Ansonia, Conn.; Gilbert Knapp Ellis, Clayton, N. Y.

The manner of conferring degrees at this institution is by "hooding," in pursuance of an ancient rite observed in many of the English universities. It is conducted as follows: The candidates, wearing their long black gowns, are introduced by a graduate to the Chancellor of the University, with the following words:

Insignissime Cancellarie.

Presento tibi huncce scholarum in facultate medicinæ ut admittatur in gradum doctoris medicina testorque eum quoad omnia quae statuta requirunt aptum et idoneum esse.

Each candidate then kneels before the Chancellor, who holds the candidate's hands in his and pronounces the following formula, during which the candidate is "hooded" by another graduate who acts as a beadle:

Ad profectum reipublicae ego, auctoritate mea et totius universitatis admitto te ad gradum doctoris in medicina licentiamque tibi do omnia ea facienda quae ad illum gradum pertinent.

Instead of the Hippocratic oath, a special formula is used, covering all the responsibilities of the profession, taking "Religio, mores, cultura" as their motto. The ceremony of conferring of degrees was performed amid applause.

Prof. A. R. Davidson, of the faculty, delivered the address to the graduates, which is found in this number of the JOURNAL.

The Rev. Dr. Dodge, President of Madison University, Hamilton, N. Y., delivered a stirring address, in the course of which he characterized the graduates as specialists. First and foremost, they were to succeed in their profession, and the man endowed with original aptitude for that profession was called of God. They should make their profession the center of their thought, of their feeling, and of their intellectual activity. Culture was governed largely by environments, by taste, and by the means at command, but first and foremost, they should resolve that, with the help of God, they would succeed. They should carry into their calling all their enthusiasm, rise to the level of their possibilities, and carry the soul-life into their work. It was true that their calling would be used as a means of support, but there were other motives; there were the inspirations of the intelligence and the heart, and the blending of these twain gave enthusiasm. The man who would reach the highest position in his profession would be he who would be best equipped, and on whose character people would rely with unfaltering trust. While they were specialists, they were not to be simply and solely specialists, confined within the narrow boundaries of their calling and vocation. The essential elements of to-day were, first, central convictions, and second, breadth of apprehension. The radius of their central convictions need not be narrow. They were to be leaders in society, but not dictators. The true leader he defined to be the man who embodied the best thought, the best sentiment, the noblest purpose in those whom he leads, presenting to men a revelation of themselves. High leadership was associated with morals and religion. In conclusion, Dr. Dodge took up an idea suggested by the remarks of the preceding speaker, and brought out with striking clearness the littleness of science and its inadequacy to solve the problem of life. His last words to the graduates were, "Be loyal to yourselves and what is deepest in you, and you will be loyal to God, who is above you, and you will be leaders in society."

THE BANQUET.

The commencement exercises over, the Faculty, Alumni, graduates, and invited guests, to the number of seventy, repaired to the Genesee, where an elaborate banquet was spread in the grand dining hall for their appreciative consumption. As is usual on such occasions, the final act in the drama of that day, which is an epoch in the lives of the graduates, proved the most enjoyable to the participants. The gastronomic portion of the feast began at 10.30, and lasted until midnight.

The edibles cleared away, the orators began. Dr. Floyd S. Crego acted as toast-master, the sentiments and respondents thereto being as follows:

"Niagara University"—Rev. Father Kavanaugh, of Niagara Falls.

"The Medical Faculty"—Dr. S. T. Clark, of Lockport.

"The Clergy"—Rev. I. N. Dalby.

"The Law"—Adelbert C. Moot.

"Dentistry"—Dr. F. E. Howard.

"Regents of the State of New York"—Hon. William Cobb, of Lockport.

"The Maternity Hospital"—Dr. Fredericks.

"The College"—Hon. T. V. Welch, of Niagara Falls.

"The Alumni of Niagara University"—Dr. E. J. Murphy.

"The Graduates"—Dr. L. G. Hanley.

The responses were all able and eloquent, some of the speeches brimming over with that rich humor which is so favorably received on occasions of this character. Musical interspersions were given by the university quartette, some of the songs being composed for the occasion.

